# Does Vitamin C Supplementation Provide a Protective Effect in Periodontal Health? A Systematic Review and Meta-Analysis

**DOI:** 10.3390/ijms25168598

**Published:** 2024-08-07

**Authors:** Roxana Buzatu, Magda Mihaela Luca, Bogdan Andrei Bumbu

**Affiliations:** 1 Department of Dental Aesthetics, Faculty of Dental Medicine, “Victor Babes” University of Medicine and Pharmacy Timisoara, Revolutiei Boulevard 9, 300041 Timisoara, Romania; roxana.buzatu@umft.ro; 2 Department of Pediatric Dentistry, Faculty of Dental Medicine, “Victor Babes” University of Medicine and Pharmacy Timisoara, Eftimie Murgu Square 2, 300041 Timisoara, Romania; 3 Department of Dental Medicine, Faculty of Medicine and Pharmacy, University of Oradea, 410073 Oradea, Romania; bogdanbumbu@uoradea.ro

**Keywords:** vitamin C, dentistry, periodontal disease, stomatology, oral health, nutritional supplementation

## Abstract

Recent research has highlighted potential benefits of vitamin C in managing periodontal diseases, yet systematic reviews to consolidate these findings are scarce. This study aims to evaluate the effectiveness of vitamin C supplementation in preventing and treating periodontal diseases and elucidate the biological mechanisms underlying these effects. We conducted a systematic review following PRISMA guidelines, searching three databases up to 13 April 2024, for studies from 2010 onward. Our selection criteria aimed to capture a wide range of studies regarding vitamin C’s impact on periodontal health. After rigorous screening, 16 studies were included in the final analysis. Meta-analysis techniques were employed to synthesize data and evaluate the association between vitamin C intake and periodontal disease outcomes. The meta-analysis included 17,853 participants from studies with diverse geographical and demographic settings. Notable findings indicated that higher vitamin C intake was associated with a reduction in periodontal disease risk, with a pooled odds ratio (OR) of 1.52 (95% CI: 1.49–1.55). The individual studies reported ORs ranging from 0.62 (95% CI: 0.38–0.94) indicating significant protective effects, to 1.66 (95% CI: 1.04–2.64), suggesting increased risks associated with inadequate vitamin C levels. The heterogeneity among the studies was high (I^2^ = 95.46%), reflecting variability in study design and population characteristics. This systematic review confirms that vitamin C supplementation has a beneficial effect on periodontal health. The significant variability across studies suggests that individual dietary needs and baseline vitamin C levels might influence the effectiveness of supplementation. These findings underscore the importance of personalized nutritional guidance as part of comprehensive periodontal care. Future research should focus on longitudinal studies to better understand the causal relationships and potential confounding factors affecting the link between vitamin C intake and periodontal health.

## 1. Introduction

Periodontal disease, encompassing a range of inflammatory conditions affecting the supporting structures of the teeth, is a major public health concern globally [1,2]. Despite advancements in dental practices, periodontitis remains highly prevalent, affecting approximately 20–50% of the global population [3,4]. The pathophysiology of periodontal disease involves complex interactions between microbial biofilms and the host’s immune response, leading to the destruction of periodontal ligament and alveolar bone [5,6]. The role of nutrition, particularly micronutrients, in the modulation of periodontal health has garnered considerable scientific interest.

Vitamin C, a potent antioxidant, is essential for the synthesis of collagen and the maintenance of connective tissue integrity, functions critical to periodontal structure and health [7,8,9,10]. The antioxidant properties of vitamin C contribute to its protective role in periodontal health by neutralizing reactive oxygen species (ROS) generated during inflammatory processes [11]. Excessive ROS can exacerbate periodontal destruction by damaging cellular structures and modifying signaling pathways that regulate inflammatory responses [12]. Moreover, vitamin C influences the immune system; it enhances the function of phagocytes and proliferation of T-lymphocytes, crucial components in the host defense against periodontal pathogens [13].

There is also emerging interest in the synergistic effects of vitamin C with other micronutrients, such as vitamin D and calcium, on periodontal health [14,15,16,17]. This synergy could potentially enhance the therapeutic outcomes of supplementation regimens, offering a multifaceted approach to managing periodontal disease. Nevertheless, the clinical application of such findings necessitates rigorous randomized controlled trials to establish clear guidelines and recommendations.

However, the oral cavity’s complexity as a system, with its unique environmental conditions and microbial interactions, poses significant challenges in isolating the effects of vitamin C. While it enhances the function of phagocytes and promotes T-lymphocyte proliferation—key defenses against periodontal pathogens—the application of vitamin C as an anti-inflammatory cofactor in clinical settings remains underexplored and not fully substantiated by clear, causal evidence [7,8,9,10,11,12,13]. This highlights the need for more targeted research to verify vitamin C’s role within this intricate biological system, especially as a potential therapeutic agent in the management of periodontal diseases.

To address the gaps in existing research, this systematic review poses specific research questions: Does regular vitamin C supplementation reduce the risk and severity of periodontal diseases? What are the mechanisms through which vitamin C exerts its protective effects on periodontal health? Furthermore, the study hypothesizes that adequate vitamin C intake, as part of a balanced diet or supplementation regimen, significantly reduces the incidence and severity of periodontal diseases by enhancing immune function and reducing oxidative stress.

Based on the current understanding of vitamin C’s role in periodontal health and the gaps identified in existing research, this systematic review aims to comprehensively assess the evidence on the effectiveness of vitamin C in the prevention and treatment of periodontal diseases. Our hypothesis is that adequate intake of vitamin C significantly reduces the risk and severity of periodontal diseases. The objectives of this study are to evaluate the impact of vitamin C supplementation on clinical periodontal outcomes across different populations and to clarify the underlying biological mechanisms through which vitamin C influences periodontal health.

## 2. Materials and Methods

### 2.1. Eligibility Criteria and Information Sources

For this systematic review, we established specific inclusion and exclusion criteria to ensure the rigorous selection of studies: (1) Studies must involve human participants diagnosed with periodontal diseases; (2) Research must explicitly examine the impact of vitamin C intake—through diet or supplementation—on periodontal health. This encompasses studies assessing clinical outcomes such as gingival inflammation, bleeding on probing, pocket depth reduction, and alveolar bone preservation; (3) The review included a broad array of study designs, such as randomized controlled trials, observational studies, cohort studies, case-control studies, and cross-sectional studies; (4) Regarding the outcome measures, the current review included studies that utilize validated instruments or clearly defined clinical parameters to assess periodontal health outcomes that consist of periodontal disease indices, biochemical markers of inflammation, and radiographic evidence of alveolar bone health; (5) Only peer-reviewed articles published in English were considered.

The exclusion criteria encompassed: (1) Non-human studies; (2) Studies that examine the impact of general multivitamin supplements or broad dietary patterns without specific focus on vitamin C were excluded; (3) Studies that do not provide clear, quantifiable outcomes related to periodontal health, or lack sufficient detail for a comprehensive analysis, were also excluded; (4) To maintain the credibility and reliability of the data included in the review, grey literature, including non-peer-reviewed articles, preprints, conference proceedings, general reviews, commentaries, editorials, systematic reviews and meta-analyses were also excluded. The exclusion of grey literature, including general reviews and commentaries, from this systematic review ensures the inclusion of only peer-reviewed studies, upholding the highest standards of scientific accuracy and reducing bias. These excluded sources often lack rigorous peer review and can introduce secondary interpretations not grounded in primary data, potentially skewing the review’s findings.

This study employed a comprehensive search strategy across three electronic databases, including PubMed, Scopus, and Web of Science. The literature search targeted publications up to 13 April 2024, and no older than 2010, capturing the most recent and relevant studies on the topic.

### 2.2. Search Strategy

The search strategy utilized a comprehensive array of keywords and phrases pertinent to the study’s objectives, focusing on the impact of vitamin C on periodontal health. Key search terms included: “vitamin C”, “ascorbic acid”, “periodontal health”, “periodontitis”, “gingivitis”, “periodontal disease”, “dental health”, “gingival health”, “oral health”, “collagen synthesis”, “antioxidant properties”, “immune response in periodontal disease”, “inflammatory response”, and “clinical outcomes in periodontal treatment”.

To ensure a comprehensive and efficient literature retrieval, Boolean operators (AND, OR, NOT) were employed to effectively combine and refine search terms. The search string included the following: ((“vitamin C” OR “ascorbic acid”) AND (“periodontal health” OR “periodontitis” OR “gingivitis”) AND (“collagen synthesis” OR “antioxidant properties”) AND (“immune response” OR “inflammatory response”) AND (“clinical outcomes” OR “treatment efficacy” OR “disease progression”)). This strategy was designed to capture the most relevant studies assessing the therapeutic impacts of vitamin C on various aspects of periodontal health.

### 2.3. Data Collection and Selection Process

This systematic review evaluates the effectiveness of vitamin C supplementation on periodontal health, focusing on individuals diagnosed with periodontal diseases (Population). The intervention in question is vitamin C supplementation, administered either through diet or direct supplementation (Intervention). This study compares the outcomes of individuals receiving vitamin C supplementation against those with no supplementation or inadequate vitamin C levels (Comparison). The primary outcomes assessed include clinical measures of periodontal health such as gingival inflammation, bleeding on probing, pocket depth reduction, and alveolar bone preservation (Outcome).

The current study followed the Preferred Reporting Items for Systematic Reviews and Meta-Analyses (PRISMA) guidelines [18]. Initially, all retrieved records were independently screened by two reviewers to determine their eligibility based on the predefined inclusion and exclusion criteria. Discrepancies between reviewers were resolved through discussion or, if necessary, consultation with a third reviewer. The review protocol, including the detailed selection methodology, has been registered and is openly accessible on the Open Science Framework (OSF) with the registration code osf.io/by274, ensuring transparency of our research process and findings.

The data collection process for this systematic review began with the removal of duplicate entries, followed by a rigorous screening of abstracts by two independent reviewers to assess each study’s relevance based on predefined inclusion and exclusion criteria. Discrepancies between reviewers were resolved through discussion or, if necessary, consultation with a third reviewer to achieve consensus. The initial database search yielded a total number of articles, which were evaluated and subsequently identified for inclusion in the final study. This process ensured a comprehensive and unbiased collection of data pertinent to the effects of vitamin C on periodontal health.

### 2.4. Data Items

The primary outcomes of interest were clinical measures of periodontal health, including gingival inflammation, periodontal pocket depth, clinical attachment loss, and alveolar bone level changes. To provide a detailed analysis of how vitamin C influences periodontal health, we also collected data on secondary outcomes related to systemic markers of inflammation and antioxidant status. Additionally, we gathered information on participant demographics (age, gender, ethnicity, smoking status), study characteristics (country, year, design, sample size), and quality of studies to assess the representativeness and applicability of the findings. Nutritional status and intake levels of other relevant micronutrients were also recorded to evaluate potential confounding factors and synergistic effects with vitamin C.

This systematic review defined periodontal health outcomes according to standardized clinical parameters commonly used in dental research, such as the Community Periodontal Index (CPI) and bleeding on probing. We included studies that provided clear definitions of these measures and whose methodologies adhered to internationally recognized standards. For each included study, we extracted data related to the type and amount of vitamin C consumed, whether through diet or supplementation, and the specific methods used to assess vitamin C intake (e.g., dietary questionnaires, blood serum levels).

### 2.5. Risk of Bias and Quality Assessment

For the systematic assessment of study quality and determination of risk of bias within the included studies, our review employed a dual approach, integrating both qualitative and quantitative evaluation methods. The quality of observational studies was evaluated using the Newcastle–Ottawa Scale [19], and Grading of Recommendations Assessment, Development, and Evaluation (GRADE) tool. Each study is awarded stars in these categories, cumulating in a score that classifies the study quality as either low, medium, or high.

For randomized controlled trials, the Cochrane Collaboration’s tool for assessing risk of bias was utilized [20]. This tool evaluates several domains including random sequence generation, allocation concealment, blinding of participants and personnel, blinding of outcome assessment, incomplete outcome data, selective reporting, and other biases. Each domain is judged as ”low risk”, ”high risk”, or ”unclear risk” of bias. To ensure the objectivity and reproducibility of our quality assessment process, each study was independently evaluated by two researchers. Discrepancies in quality assessment scores were resolved through discussion, or if necessary, consultation with a third researcher.

### 2.6. Synthesis Methods

In this systematic review, we synthesized findings from selected studies through a qualitative and quantitative synthesis, focusing on the role of vitamin C in periodontal health. The selection of studies for synthesis was based on their alignment with predefined inclusion criteria, specifically those studies that provided data on vitamin C intake and its impact on clinical periodontal outcomes such as pocket depth, bleeding on probing, and clinical attachment loss. To prepare data for synthesis, we tabulated outcomes related to periodontal health improvements, inflammatory markers, and vitamin C intake levels, while noting any missing data and acknowledging potential impacts on our findings. Results from individual studies were summarized and presented descriptively, comparing periodontal health outcomes across diverse geographic and clinical settings.

A meta-analysis was performed to evaluate the effectiveness of vitamin C supplementation or dietary intake on specific periodontal health outcomes. Heterogeneity among study results was quantified using the I^2^ statistic, which describes the percentage of total variation across studies that is due to heterogeneity rather than chance. A high I^2^ value indicates substantial variability among the studies. All analyses were performed using standard statistical software, ensuring that all estimates were accompanied by 95% confidence intervals to assess the precision of the findings.

## 3. Results

### 3.1. Study Selection and Study Characteristics

**Figure 1:**
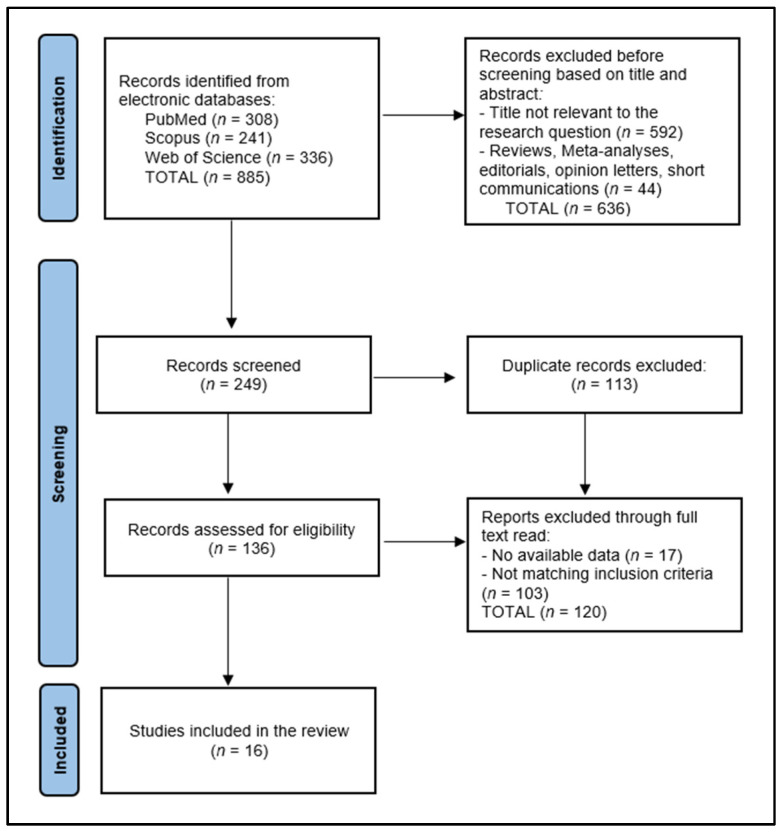
PRISMA Flow Diagram.

A total of 885 articles were identified according to the initial search (308 in PubMed, 241 in Scopus, and 336 in Web of Science), of which 113 duplicate entries were eliminated, 636 records excluded before screening based on title and abstract, and 120 articles excluded after full read for not matching the inclusion criteria or having no available data (Figure 1). The systematic review included a total of 16 eligible studies in the final analysis, as presented in Table 1.

### 3.2. Results of Individual Studies

The individual studies included in this meta-analysis examined the impact of vitamin C on periodontal disease across a diverse set of populations and age groups, offering a rich dataset spanning various global regions. The studies included a total of 17,853 participants, with individual study sizes ranging from 21 participants in Kuzmanova et al. [27] to 12,980 participants in Lee et al. [22], reflecting both small-scale community studies and larger, more extensive research efforts. Age distributions varied significantly, with Hosoda et al. [21] focusing on a remarkably young cohort with an average age of 20.4 years, while Iwasaki et al. [28] targeted a much older population, over 75 years, as presented in Table 2. The gender distribution across these studies also varied, although several studies showed a female predominance or nearly equal gender distribution, which is somewhat atypical in periodontal disease studies where male prevalence is often higher.

### 3.3. Results of Synthesis

Notably, the studies by Lee et al. [22] and Li et al. [23] featured large sample sizes and reported moderate effect sizes with ORs of 1.16 and 1.13, respectively, suggesting a consistent but moderate protective effect of adequate vitamin C intake against periodontal disease. On the other hand, Park et al. [25] and Luo et al. [24] reported higher odds ratios (1.66 and 1.40), indicating a stronger association in specific populations. Kuzmanova et al. [27] and Iwasaki et al. [28] provided evidence of a significant protective effect of high vitamin C intake with odds ratios substantially below 1 (0.62 and 0.72), highlighting the potential for dietary vitamin C to mitigate periodontal disease risks significantly. Lastly, Yoshihara et al. [34] found that low serum vitamin C levels were associated with an increased risk of periodontal attachment loss (OR = 1.58), reinforcing the role of nutritional status in periodontal health (Table 3).

**Figure 2:**
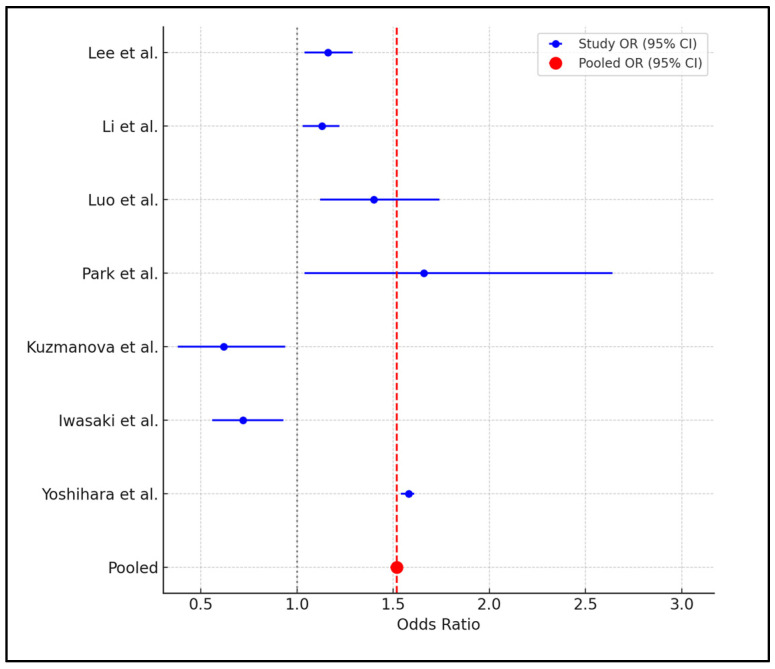
Forest plot of meta-analysis [22,23,24,25,27,28,34].

The meta-analysis of seven studies examining the relationship between vitamin C intake and periodontal health yielded a pooled odds ratio (OR) of 1.52, with a narrow 95% confidence interval (CI) of 1.49 to 1.55, indicating a significant association between higher vitamin C intake and improved periodontal outcomes. However, the high heterogeneity (I^2^ = 95.46%) among the studies suggests considerable variability in how vitamin C affects periodontal health across different populations or under different study conditions (Figure 2).

## 4. Discussion

The studies incorporated into the meta-analysis reflect a significant diversity in methodology and demographics, showcasing the complex interaction between vitamin C intake and periodontal health. Notably, studies with larger cohorts such as those conducted by Lee et al. [22] and Li et al. [23] suggested a consistent yet moderate protective effect of adequate vitamin C intake. These findings were further supported by Park et al. [25] and Luo et al. [24], who reported stronger associations between inadequate vitamin C levels and increased periodontal disease risk. Such results underscore the potential of dietary modification as a part of preventive dental care, particularly in reducing the risk of periodontal diseases.

Conversely, Kuzmanova et al. [27] and Iwasaki et al. [28] demonstrated that higher dietary vitamin C intake could significantly mitigate periodontal disease risks, with odds ratios substantially below one (0.62 and 0.72, respectively). These studies highlight the potential therapeutic benefits of vitamin C in maintaining periodontal health and suggest that supplementation could be particularly effective in populations at higher risk of periodontal diseases. Furthermore, Yoshihara et al. [34] provided compelling evidence linking low serum vitamin C levels to increased risks of periodontal attachment loss, reinforcing the importance of adequate nutritional intake in periodontal disease prevention.

The observed high heterogeneity (I^2^ = 95.46%) among the included studies is indeed noteworthy and merits thorough investigation to identify potential sources. This variability could stem from several factors, including differences in study populations, such as variations in baseline nutritional status, demographic characteristics, or the severity of periodontal disease. Intervention disparities, such as the dosage and form of vitamin C supplementation, along with differences in study designs and outcome measures, may also contribute significantly to this heterogeneity.

In recent studies examining the impact of vitamin C on periodontal ligament cells (PDLCs), distinct protective mechanisms against periodontal degradation have been highlighted. The work by Yan et al. [37] elucidated that vitamin C not only promotes the osteogenic differentiation of PDL progenitor cells but does so via specific molecular pathways. Specifically, they demonstrated that vitamin C activates the ERK pathway, which in turn up-regulates PELP1 expression and facilitates the expression of the osteogenesis marker Runx2. This finding suggests a targeted molecular mechanism whereby vitamin C could be utilized in regenerative medicine specifically tailored for periodontal disease treatment, leveraging the PELP1-ERK axis for bone and tissue regeneration. Meanwhile, Wu et al. [38] explored the antioxidative role of vitamin C against oxidative stress in PDLCs induced by hydrogen peroxide, a common byproduct of inflammation in periodontitis. Their study showed that vitamin C significantly mitigated the cytotoxic effects of hydrogen peroxide by reducing apoptosis in PDLCs, as evidenced by decreased activation of apoptosis markers such as caspases-3, caspases-9, and poly (ADP-ribose) polymerase. These findings highlight vitamin C’s potential as a therapeutic agent that not only counters oxidative stress but also supports cellular survival and function in the inflammatory environments typical of periodontal diseases. Older studies such as those by Amarasena et al. [39] and Ekuni et al. [40] also found interesting outcomes after vitamin C use in periodontal health. In the study by Amarasena et al. [39], an investigation into the serum vitamin C levels and periodontal health among elderly Japanese citizens revealed a statistically significant but modest inverse relationship between serum vitamin C concentration and clinical attachment loss (CAL). Specifically, the study found that CAL was 4% greater in subjects with lower serum vitamin C levels, even after accounting for variables such as smoking, diabetes, oral hygiene, gender, and the number of teeth present (r = −0.23, *p* < 0.00005). Conversely, Ekuni et al. [40] explored the effects of vitamin C on oxidative stress and atherosclerosis in a rat model with ligature-induced periodontitis. They demonstrated that vitamin C supplementation significantly reduced oxidative markers and lipid deposition in the aorta, thus attenuating the progression of atherosclerosis triggered by periodontal disease. Notably, vitamin C intake increased plasma vitamin C levels and the GSH:GSSG ratio by 178% and 123%, respectively, while decreasing serum hexanoyl-lysine (HEL) and aortic levels of nitrotyrosine, HEL, and 8-hydroxydeoxyguanosine by 23%, 87%, 84%, and 38%, respectively.

Other older studies explored the relationship between vitamin C levels and periodontal disease, offering insights into how nutritional status impacts periodontal health. Timmerman et al. [41] found a modest negative correlation (r = −0.199, *p* < 0.05) between plasma vitamin C levels and periodontal attachment loss among subjects from a tea estate in Indonesia, revealing that low vitamin C levels explained 3.9% of the variance in periodontal attachment loss. Notably, subjects with vitamin C deficiency experienced significantly more attachment loss compared to those with normal levels, underscoring the potential role of vitamin C in mitigating periodontal breakdown. Conversely, Pussinen et al. [42] demonstrated a significant relationship between low plasma vitamin C concentrations and higher serological markers for *P. gingivalis*, a key pathogen in periodontitis. This correlation remained significant even after adjusting for confounding factors, indicating that low vitamin C levels might enhance the colonization or impact the healing processes related to *P. gingivalis* infections. Collectively, these findings emphasize the importance of adequate vitamin C intake in maintaining periodontal health and potentially moderating the pathogenicity of periodontal infections.

The clinical implications of these findings are profound. Regular monitoring and enhancement of dietary vitamin C intake could be recommended as part of routine dental care, especially for individuals at elevated risk of periodontal diseases. Dental professionals might consider dietary assessments and vitamin C supplementation as adjunctive strategies to conventional periodontal treatments, particularly for patients showing early signs of periodontal diseases or those who are at a higher risk due to systemic health conditions. Overall, these findings advocate for a more integrated approach in dentistry that combines nutritional management with traditional periodontal therapies to optimize patient outcomes.

Although a significant risk was identified with low vitamin C levels or insufficient vitamin C supplementation in periodontal disease, there was a high heterogeneity among studies. This variability limits the study findings, and it could be attributed to differences in study designs, population characteristics, definitions of periodontal health, and methods of assessing vitamin C intake. Notably, studies showing both protective effects (Kuzmanova et al. [27], Iwasaki et al. [28]) and risk associations (Lee et al., Li et al., Luo et al., Park et al., Yoshihara et al. [22,23,24,25,34]) highlight the complex role of vitamin C in periodontal health, underscoring the need for personalized dietary recommendations and further research to clarify these relationships.

This study aligns with recent findings that highlight the role of vitamin C in directing gene differentiation pathways at the stem cell level, which could be pivotal for future regenerative therapies. The ability of ascorbic acid to activate specific molecular pathways, such as the ERK pathway observed in periodontal ligament cells, underlines its potential to influence stem cell plasticity and differentiation orientation, enhancing the therapeutic outcomes in tissue engineering and regenerative medicine [43].

The manuscript highlights the potential benefits of vitamin C supplementation for periodontal health. To better translate these findings into clinical practice, we recommend that dental practitioners advise patients on optimal vitamin C intake levels based on current dietary guidelines, which suggest a daily intake of 65 to 90 milligrams for adults, depending on age and sex. For patients with or at risk of periodontal disease, a higher intake, tailored to individual health needs and confirmed by a healthcare provider, may be beneficial. Additionally, we suggest integrating discussions about dietary sources rich in vitamin C, such as citrus fruits and leafy greens, during dental consultations.

The call for further research into the role of vitamin C in periodontal health should be emphasized by suggesting specific study designs that could provide more definitive evidence. We recommend conducting longitudinal studies and randomized controlled trials to ascertain causality and the effectiveness of vitamin C supplementation over time. Future research should also aim to include diverse populations to explore the variability in response based on genetic, dietary, and environmental factors. Addressing these gaps, such as the impact of different forms of vitamin C (e.g., from natural sources versus supplements) and the interaction with other micronutrients, will enhance our understanding and inform more effective, personalized periodontal treatment protocols.

This study acknowledges certain inherent limitations in its design, such as to limit the search to studies published from 2010 onwards was aimed at ensuring the inclusion of contemporary research with standardized definitions and outcome measures for periodontal disease. While this approach provided a focused review of recent findings, it may have excluded relevant historical data and findings reported in other databases or journals.

## 5. Conclusions

This systematic review and meta-analysis supports the association between adequate vitamin C intake and a decreased risk of periodontal diseases, with a pooled odds ratio of 1.52 suggesting a positive correlation. However, the considerable heterogeneity observed across the studies, as indicated by an I^2^ of 95.46%, necessitates a cautious interpretation of these results. This variability may stem from differences in dietary behavior, baseline nutritional status, and methodological discrepancies between studies. Given these findings, while there is evidence supporting the benefit of vitamin C, the results underscore the importance of considering individual dietary needs when making nutritional recommendations for the prevention and management of periodontal diseases. Further research is essential to more definitively determine the mechanisms by which vitamin C impacts periodontal health and to assess the efficacy of vitamin C supplementation in diverse clinical scenarios and populations.

## Figures and Tables

**Table 1:** Study characteristics.

Study and Author	Country	Study Year	Study Design	Study Quality
[21] Hosoda et al.	Japan	2020	Cross-sectional	Medium
[22] Lee et al.	South Korea	2017	Cross-sectional	Medium
[23] Li et al.	China	2022	Cross-sectional	High
[24] Luo et al.	China	2018	Cross-sectional	High
[25] Park et al.	South Korea	2017	Cross-sectional	High
[26] Watson et al.	United Kingdom	2022	Cross-sectional	High
[27] Kuzmanova et al.	The Netherlands	2012	Cross-sectional	Medium
[28] Iwasaki et al.	Japan	2012	Retrospective cohort	Medium
[29] Shimabukuro et al.	Japan	2015	Randomized trial	High
[30] Gokhale et al.	India	2013	Randomized trial	Medium
[31] Sulaiman et al.	Syria	2010	Randomized trial	Medium
[32] Munday et al.	Australia	2020	Cross-sectional	Medium
[33] Assaf et al.	Palestine	2022	Cross-sectional	Medium
[34] Yoshihara et al.	Japan	2022	Cross-sectional	High
[35] Amaliya et al.	The Netherlands	2015	Prospective cohort	High
[36] Güner et al.	Turkey	2023	Cross-sectional	Medium

**Table 2:** Patients’ background characteristics.

Study and Author	Number of Participants	Comparison Group	Age (Category/Mean/Median)	Gender
[21] Hosoda et al.	49 with PD	71 without PD	20.4 years	Female: 100%
[22] Lee et al.	3812 with PD	7118 without PD	112 (2.9%) 19–29 years; 482 (12.6%) 30–39 years; 881 (23.1%) 40–49 years; 930 (24.4%) 50–59 years; 860 (22.6%) 60–69 years; 547 (14.4%) ≥ 70 years	Female: 44.2%
[23] Li et al.	4466 with PD	4493 without PD	56.7 years	Female: 51.2%
[24] Luo et al.	2274 with moderate PD, 676 with severe PD	3465 without PD or mild PD	55.3 years: moderate PD; 54.5 years severe PD	Female: 45.9% moderate PD; 29.6% severe PD
[25] Park et al.	279 with PD	1770 without PD	33.0 years	Female: 47.7%
[26] Watson et al.	1634 with PD	7842 with PD	56.4 years	Female: 55.9%
[27] Kuzmanova et al.	21 with PD	21 without PD	46.9 years	Female: 52.0%
[28] Iwasaki et al.	264 with PD	NR	>75 years	Female: 46.6%
[29] Shimabukuro et al.	150 with vitamin C supplementation (dentifrice)	150 without vitamin C supplementation (dentifrice)	38.1 years	Female: 48.0%
[30] Gokhale et al.	90 with PD and	30 without PD	30–60 years	NR
[31] Sulaiman et al.	30 with PD (15 with vitamin C supplementation, 15 without vitamin C supplementation)	30 without PD	41.0 years	Female: 70.0%
[32] Munday et al.	20 with PD	NR	65.0 years	Female: 50.0%
[33] Assaf et al.	25 with PD	NR	55.0 years	Female: 48.0%
[34] Yoshihara et al.	353 with PD	NR	70.0 years	Female: 45.6%
[35] Amaliya et al.	98 with PD and vitamin C supplementation for 90 days (60 mg/day)	NR	33–43 years	NR
[36] Güner et al.	25 with PD	24 without PD	42.5 years	Female: 52.0%

PD—Periodontal Disease; NR—Not Reported.

**Table 3:** Assessment of periodontal disease risk.

Study and Author	Periodontal Disease Characteristics	Diabetes/Smoking	Vitamin C Assessment	Risk Assessment (OR/HR/RR—95% CI)
[21] Hosoda et al.	Periodontal pocket > 4 mm	NR	48 mg/1000 kcal (PD group) vs. 52 mg/1000 kcal (non-PD group)	NR
[22] Lee et al.	Periodontal pocket > 3.5 mm (CPI scores 3–4)	Diabetes 13.4% (PD group) vs. 6.2% (non-PD group) *	Inadequate dietary vitamin C 48.1% (PD group) vs. 45.2% (non-PD group) *; Quartile of vitamin C in the diet 23.2% ≥ 132.22 mg/day (PD group) vs. 25.9% ≥ 132.22 mg/day (non-PD group) *	Inadequate dietary vitamin C OR = 1.16 (95% CI = 1.04–1.29) * for periodontal disease
[23] Li et al.	Severe/moderate periodontitis defined as ≥2 Interproximal sites with CAL of ≥4 mm or pocket depth of ≥5 mm	Diabetes 21.2% (PD group) vs. 11.2% (non-PD group) *; Smoking 24.3% (PD group) vs. 12.6% (non-PD group) *	Inadequate dietary vitamin C 58.0% (PD group) vs. 55.8% (non-PD group)	Vitamin C > 90 mg/day in men OR = 1.13 (95% CI = 1.03–1.22) * for periodontal disease
[24] Luo et al.	Severe/moderate periodontitis defined as ≥2 Interproximal sites with CAL of ≥4 mm or pocket depth of ≥5 mm	Diabetes 15.4% (moderate PD group) vs. 13.6% (severe PD group) vs. 7.0% (non-PD group) *; Smoking 29.8% (moderate PD group) vs. 27.4% (severe PD group) vs. 25.1% (non-PD group) *	Quartile of vitamin C in the diet 25.1% ≥ 112.91 mg/day (moderate PD group) vs. 22.4% ≥ 112.91 mg/day (severe PD group) 25.5% ≥ 112.92 mg/day (non-PD group) *	Inadequate dietary vitamin C OR = 1.40 (95% CI = 1.12–1.74) * for periodontal disease
[25] Park et al.	Periodontal pocket > 3.5 mm (CPI scores 3–4)	Diabetes 1.4% (PD group) vs. 1.3% (non-PD group); Smoking 37.3% (PD group) vs. 24.7% (non-PD group) *	Inadequate dietary vitamin C (<100 mg/day) 63.1% (PD group) vs. 59.8% (non-PD group) *	Inadequate dietary vitamin C OR = 1.66 (95% CI = 1.04–2.64) * for periodontal disease
[26] Watson et al.	Self-reported questionnaire about painful gums, bleeding gums, or loose teeth	NR	NR	Vitamin C >100 mg/day in men OR = 0.81 (95% CI = 0.70–0.96)
[27] Kuzmanova et al.	Radiographic bone loss > 1/3 of the root length at ≥1 tooth per quadrant and ≥20 teeth	Smoking 52% (PD group) vs. 52% (non-PD group)	Vitamin C supplements: 14% (PD group) vs. 10% (non-PD group); Plasma vitamin C: 8.3 mg/L (PD group) vs. 11.3 mg/L (non-PD group) *	Adequate dietary vitamin C OR = 0.62 (95% CI = 0.38–0.94) * for periodontal disease
[28] Iwasaki et al.	CAL of ≥3 mm or pocket depth of ≥3 mm	Diabetes 8.3%; Smoking 47.7%	Median 91.8 mg/day	Adequate dietary vitamin C OR = 0.72 (95% CI = 0.56–0.93) * for periodontal disease
[29] Shimabukuro et al.	GI at 3 months 0.73 (dentifrice group) vs. 0.84 (control group) *; GSI at 3 months 0.21 (dentifrice group) vs. 0.15 (control group)	Smoking 18.7% (dentifrice group) vs. 20.0% (no dentifrice group)	NR	NR
[30] Gokhale et al.	SBI score of ≥2	Diabetes 0.0% (excluded); Smoking: 0.0% (excluded)	450 mg daily ascorbic acid supplementation; Sulcus bleeding index difference 0.56 (vitamin C supplementation) vs. 0.28 (no vitamin C supplementation) *	NR
[31] Sulaiman et al.	GI > 3.5 mm and CAL > 3.5 mm	NR	Plasma TAOC levels 625 mm Teq (without PD) vs. 559 mm Teq (with PD) *	NR
[32] Munday et al.	Radiographic bone loss around teeth in the coronal third, extending to the mid third of the root or beyond	Diabetes 20.0%; Smoking 20.0%	Vitamin C < 40 µmol/L: 30.0% of patients; CRP levels > 4 umol/L 25.0% of patients—significant inverse correlation between vitamin C levels and CRP	NR
[33] Assaf et al.	NR	NR	40% of patients with low vitamin C < 40 µmol/L had stage IV PD *	NR
[34] Yoshihara et al.	PAD ≥ 4 mm and CAL ≥ 4 mm	Smoking 100%	Serum vitamin C levels (mean): 7.36 ug/mL	Low serum vitamin C levels OR = 1.58 (95% CI = 1.54–1.61) for CAL ≥ 4 *
[35] Amaliya et al.	NR	Diabetes 6.1%	Serum vitamin C levels (mean): 5.19 mg/L, Optimal vitamin C levels (>8.8 mg/L) in 13.3% of patients	Supplementation with VitC/Ca/F reduced the subgingival load of all studied bacteria
[36] Güner et al.	In all groups, periodontal status was evaluated with plaque index and GI	NR	Serum vitamin C levels (mean): 7.00 mg/L (PD group) vs. 8.83 (non PD group) *; Vitamin C intake 124.6 mg/day (PD group) vs. 176.7 mg/day (non-PD group) *	The plasma ascorbic acid levels and total oxidant status (r = −0.42) and superoxide radical levels (r = −0.53) were inversely correlated in patients with periodontitis

*—statistically significant values; NR—Not Reported; OR—Odds Ratio; RR—Risk Ratio; PD—Periodontal Disease; CPI—Community Periodontal Index; CAL—Clinical Attachment Loss.

## Data Availability

Not applicable.
